# Health-Related Quality of Life in Patients with Philadelphia-Negative Myeloproliferative Neoplasms: A Nationwide Population-Based Survey in Denmark

**DOI:** 10.3390/cancers12123565

**Published:** 2020-11-28

**Authors:** Nana Brochmann, Esben Meulengracht Flachs, Anne Illemann Christensen, Marie Bak, Christen Lykkegaard Andersen, Knud Juel, Ann-Dorthe Zwisler

**Affiliations:** 1 Department of Hematology, Zealand University Hospital, 4000 Roskilde, Denmark; mabak@regionsjaelland.dk or; 2 Department of Occupational and Environmental Medicine, Bispebjerg University Hospital, 2400 Copenhagen, Denmark; esben.meulengracht.flachs@regionh.dk; 3 National Institute of Public Health, University of Southern Denmark, 1455 Copenhagen, Denmark; ach@si-folkesundhed.dk (A.I.C.); kj@si-folkesundhed.dk (K.J.); 4 Department of Hematology, University Hospital of Copenhagen at Rigshospitalet, 2100 Copenhagen, Denmark; christen.andersen@sund.ku.dk; 5 Danish Knowledge Centre for Rehabilitation and Palliative Care, University of Southern Denmark and Odense University Hospital, 5800 Nyborg, Denmark; ann.dorthe.olsen.zwisler@rsyd.dk

**Keywords:** myeloproliferative neoplasm, health-related quality of life, symptoms, fatigue, symptom burden, functioning, lifestyle, patient-reported outcome

## Abstract

**Simple Summary:**

The aim of this research was to investigate the health-related quality of life (HRQoL) in patients with Philadelphia-negative myeloproliferative neoplasms (chronic blood cancers) in Denmark. A nationwide questionnaire survey covering functioning, symptom burden, symptom profile, QoL, and lifestyle was performed. Patients registered in the National Patient Register with a diagnosis of Philadelphia-negative myeloproliferative neoplasm were invited. A total of 2228 patients participated in the survey and these could be divided into groups of participants with different subtypes of Phildelphia-negative myeloproliferative neoplasms. The HRQoL across groups of participants with different subtypes of the disease was compared, and the HRQoL of all participants and the general population was compared in order to investigate for a potential difference. The participants reported their HRQoL to be inferior to the general population, but the difference was minor. The differences in HRQoL across groups of participants with different subtypes of the disease were subtle. Fatigue and sexual problems were prevalent and burdensome. Participants reported a slightly healthier lifestyle than the general population. Understanding HRQoL of these patients is a necessity to be able to provide the best treatment and rehabilitation activities.

**Abstract:**

Previous studies have clarified that many patients with Philadelphia-negative myeloproliferative neoplasms (MPNs) have burdensome symptom profiles and accordingly impaired functioning and quality of life (QoL). In Denmark, all MPN patients are affiliated with public hospitals and because of a healthcare system financed by taxpayers these patients do not have any financial costs related to the hematological disease. Diagnoses are recorded for all patients in hospitals, and diagnosis codes are communicated to the National Patient Register (NPR). Owing to this, it was possible to contribute to the elucidation of Philadelphia-negative MPN patients’ health-related quality of life (HRQoL), by conducting a nationwide, population-based, cross-sectional HRQoL survey of these patients with cost-free access to the best available, suitable medical treatment. The survey contained validated questionnaires covering functioning, symptom burden, symptom profile, QoL, and additional questions on lifestyle. Information on comorbid diagnoses was obtained from the NPR. The participants’ HRQoL was compared to the general population. Moreover, differences in HRQoL across essential thrombocythemia, polycythemia vera, myelofibrosis, and unclassifiable MPN participants were investigated, adjusted for age, sex, comorbidity, and lifestyle. To the best of our knowledge this is the first survey of HRQoL in patients with unclassifiable MPN. A total of 2228 Philadelphia-negative MPN patients participated. The participants reported their HRQoL to be inferior to the general population, but the difference was minor. The differences in HRQoL across groups of participants with different MPN subtypes were subtle. Fatigue and sexual problems were prevalent and burdensome. Overall, participants reported a slightly healthier lifestyle compared to the general population.

## 1. Introduction

The classical Philadelphia-negative myeloproliferative neoplasms (MPNs) comprise three main diseases: essential thrombocythemia (ET), polycythemia vera (PV), and myelofibrosis (MF). Furthermore, some MPNs cannot be subclassified (MPN-U) [1,2,3,4]. Focus is increasing on disease-associated symptoms and the extent to which these affect the patients’ daily functioning and quality of life (QoL) [5,6,7,8,9,10,11,12,13,14,15,16,17,18,19,20,21,22,23,24,25,26,27]. Previous studies have clarified that many Philadelphia-negative MPN patients are burdened by symptoms [5,6,7,8,9,10,11,12,13,14,15,16,17,18,19,20,21,22,23,24,25,26,27], and symptoms have been shown to reduce daily activity, work productivity, and QoL [8]. Fatigue has been found to be the most prevalent and dominant symptom [5,6,7,8,9,10,11,12,13,14,15,16,17,18,19,20,21,22,23,24,25,26,27]. However, so far, a nationwide, population-based health-related quality of life (HRQoL) survey in MPN patients with cost-free access to best available, suitable medical treatment has not been undertaken. 

In Denmark, all MPN patients are affiliated with public hospitals and owing to a health care system financed by taxpayers, these patients are offered the best available, suitable medical treatment according to nationally applicable guidelines without any financial costs related to the treatment of the hematological disease. Diagnoses for all patients are recorded at the hospital, and diagnosis codes have been passed on and permanently saved in the National Patient Register (NPR) since 1977 [28]. At the date of birth, an inhabitant receives a Civil Personal Registration number (CPR-number), and information such as postal address is continuously updated in the Civil Personal Register (CPR) [29]. This equipped us to conduct a nationwide, population-based, cross-sectional HRQoL survey of MPN patients with equal access to the best available medical treatment (the MPNhealthSurvey) [30]. In order to examine the impact of MPN on HRQoL, we adjusted for potential confounders and these were based on information from the CPR and NPR with the exception of lifestyle reported by the participants. 

In this article, we present Philadelphia-negative MPN participants’ functioning, symptom burden, symptom profile, QoL, comorbidity, and lifestyle. The participants’ HRQoL was compared to the HRQoL of the general population. We present the results of an examination of potential differences in HRQoL among ET, PV, MF, and MPN-U participants adjusted for age, sex, comorbidity, and lifestyle. 

First, a published article presents the association between fatigue, physical activity, and QoL in ET, PV, and MF participants from the MPNhealthSurvey [31]. Second, a published article presents the prevalence and severity of anxiety and depression and the association of anxiety and depression with different factors in ET, PV, MF, and MPN-U participants from the MPNhealthSurvey [32]. Third, a published article presents the association between body mass index (BMI) and total symptom burden and symptom severity by BMI in ET, PV, MF, and MPN-U participants from the MPNhealthSurvey and the international Fatigue Study combined [33]. 

## 2. Results

### 2.1. Participants

**Figure 1:**
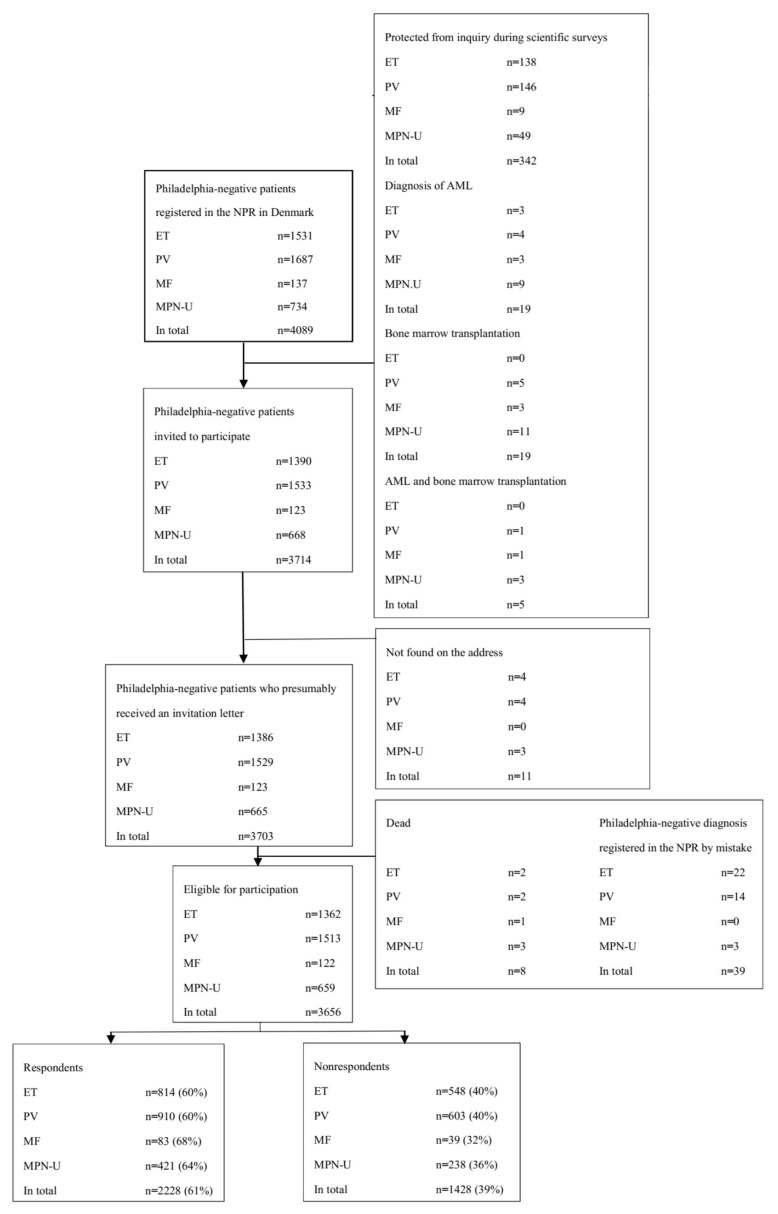
Flowchart for the survey population.

In total, 3656 individuals had a recording of an ET, PV, MF, or MPN-U diagnosis in the NPR between 1977 and 31 March 2013, fulfilled all of the inclusion criteria, and were not registered with an AML diagnosis or registered as having undergone bone marrow transplantation on 4 September 2013. Moreover, none of these individuals were listed as protected from inquiry regarding a scientific survey. Of those, 2228 (61%) participated in the survey (Figure 1). 

The mean age of the participants was 71 years and participants included 57% females and 43% males (Table 1). Among patients aged 70–79, 68% of the females and 67% of the males responded to the survey, and this was the age group with the highest response rate. The response rate among females was the same among patients aged 70–79 and 60–69. Below the age of 60, decreasing age was related to a lower response rate both for females and males. Of the participants, 66% were living with someone and 66% had completed higher education or attended an upper secondary or vocational school. Information was missing for 1% with regard to living arrangement and for 4% with regard to education. Among participants, 71% were retired, 27% were employed, and 2% were unemployed. Information was missing for one participant with regard to employment status. Of the participants, 95% were of Danish/Western ethnicity, 4% of non-Western ethnicity, and information was missing for 1%. The majority of the participants had CCI ≤ 2. Patients who were diagnosed with MPN more than 5 years before the start of the survey represented 57% while patients who were diagnosed less than 1 year before the start of the survey represented only 7% of the participants. The participants included 814 ET, 910 PV, 83 MF, and 421 MPN-U patients.

### 2.2. Missing Survey Data

A total of 77% of the participants completed the Short Form 36 Health Survey (SF-36) [34], 91% the European Organisation for Research and Treatment of Cancer Quality of Life Questionnaire Core 30 (EORTC QLQ C-30) [35], 89% the Myeloproliferative Neoplasm Symptom Assessment Form (MPN-SAF) [26], 93% the Brief Fatigue Inventory (BFI) [36], 88% the Multidimensional Fatigue Inventory (MFI) [37], and ≥94% submitted answers to the questions regarding lifestyle.

### 2.3. Functioning

The MF and MPN-U participants’ physical functioning was moderately impaired compared to the general population, whereas the ET and PV participants’ physical functioning was slightly impaired compared to the general population (ET: 77.2, PV: 73.7, MF: 70.0, MPN-U: 71.8, GP: 86, *p* < 0.001) (Table 2). The role, cognitive, and social functioning were generally slightly impaired in the MPN participants compared to the general population. The difference in emotional functioning between the MPN participants and the general population was trivial. The differences in functioning between the MPN subgroups were trivial with the exception of the ET participants having a slightly higher level of physical functioning compared to the MF and MPN-U participants, respectively [38,39,40].

### 2.4. Symptom Burden

The differences in total symptom burden among the MPN subgroups with different MPN subtypes were subtle (ET: 17.5, PV: 17.8, MF: 16.2, *p* < 0.001) (Table 3). The MPN-U participants had the highest total symptom burden relative to the classified MPN participants (MPN-U: 21.1). Among the ET, PV, and MPN-U participants fatigue had the highest prevalence compared to other symptoms (ET: 82%, PV: 82%, MPN-U: 87%). Abdominal discomfort and early satiety, dizziness, insomnia, night sweats, sexual problems, and sad mood were other symptoms with a high prevalence in all MPN subgroups. Sexual problems were reported with a relatively high mean score in all MPN subgroups (ET: 3.3, PV: 3.3, MF: 3.8, MPN-U: 3.8), and among the MF participants sexual problems had the highest prevalence compared to other symptoms (MF: 82%).

### 2.5. Fatigue

The MF and MPN-U participants reported the burden of fatigue to be moderately higher and ET and PV slightly higher compared to the general population (ET: 34.0, PV: 35.9, MF: 38.5, MPN-U: 37.4, GP: 24, *p* < 0.001) (Table 2). The differences in fatigue burden across the MPN subgroups were trivial [38,39,40]. 

The MF and ET participants reported the most and least severe general fatigue, respectively, among the classified MPN participants (ET: 11.5, PV: 11.6, MF: 12.4, *p* < 0.001) (Table 4). MPN-U participants reported general fatigue to be slightly less burdensome than the MF participants (MPN-U: 12.0). The MF participants reported the most severe and the ET participants the least severe physical fatigue among the classified participants (ET: 11.3, PV: 12.0, MF: 12.2, *p* < 0.001). Conversely, the MF and PV participants reported the least and worst mental fatigue, respectively (ET: 8.6, PV: 8.7, MF: 8.2, *p* < 0.001). The MPN-U participants reported physical fatigue to be slightly less severe than the MF participants (MPN-U: 12.1) and mental fatigue to be more severe than the classified participants (MPN-U: 8.9). Overall, the differences between the MPN subgroups in terms of different aspects of fatigue were minor.

### 2.6. Global Health/Quality of Life

MPN participants reported a slightly impaired global health/QoL compared to the general population (ET: 68.5, PV: 67.1, MF: 67.6, MPN-U: 64.9, GP: 73, *p* < 0.001) (Table 2). The differences in global health/QoL across the MPN subgroups were trivial (*p* = 0.002) [38,39,40]. 

The physical component summary measure was the lowest in MF participants and the highest in ET participants when the classified MPN subgroups were compared (ET: 47.7, PV: 46.5, MF: 45.0, *p* < 0.001) (Table 2). The physical component summary measure was marginally higher in the MPN-U participants than in the MF participants (MPN-U: 45.6). When the classified MPN participants were compared, the mental component summary measure was the lowest in PV participants and the highest in MF participants (ET: 51.5, PV: 51.3, MF: 51.9, *p* = 0.002). The mental component summary measure was lower in MPN-U participants than in participants with classified subtypes (MPN-U: 50.7). Overall, the differences in physical and mental component summary measure across the ET, PV, MF, and MPN-U participants were modest [38,39,40].

### 2.7. Comorbidity and Lifestyle

The MF and ET participants had the highest and lowest CCI, respectively (*p* < 0.0001) (Table 5). There was no significant difference in BMI among the MPN subgroups; however, ET, PV, and MPN-U participants had a significantly lower BMI than the general population (ET: *p* = 0.0011, PV: *p* = 0.0004, MPN-U: *p* = 0.029). There was neither a significant difference in the distribution of smokers, ex-smokers, and non-smokers among the MPN subgroups, nor when the MPN subgroups were compared to the general population. However, there were more ex-smokers in the MPN population than the general population. Except for MF participants, the MPN subgroups had a lower alcohol intake compared to the general population, with significant difference for the PV and MPN-U participants (PV: *p* = 0.01, MPN-U: *p* = 0.0017). There was also a significant difference among the MPN subgroups in being physically active (*p* = 0.0032). ET participants were the most and MF participants the least physically active. The MPN subgroups were more physically active than the general population (ET: *p* < 0.0001, PV: *p* < 0.0001, MF: *p* = 0.0040, MPN-U: *p* < 0.0001). However, a greater number of MF participants were completely inactive compared to the general population (MF: 39%, GP: 26%).

## 3. Discussion

The MPNhealthSurvey was a nationwide, population-based, cross-sectional survey of HRQoL in MPN patients in Denmark [30]. A total of 2228 (61%) Philadelphia-negative classified and unclassifiable MPN patients responded to the survey. The most striking findings from the investigation of HRQoL in Philadelphia-negative MPN patients and comparison with the general population are the small difference in HRQoL between the MPN population and the general population and the subtle differences in HRQoL between the MPN subgroups with different MPN subtypes. Difference in functioning between the MPN population and the general population was small with the exception of MF and MPN-U participants, respectively, reporting physical functioning to be moderately impaired compared to the general population and the difference in emotional functioning between the MPN population and general population being trivial. The difference in functioning and total symptom burden across the MPN subgroups was trivial except for physical functioning being slightly more impaired in MF and MPN-U participants than in ET and PV participants. Fatigue and sexual problems were prevalent and burdensome. The difference in fatigue across the MPN subgroups was subtle, but fatigue was moderately more burdensome in MF and MPN-U and slightly more burdensome in ET and PV participants compared to the general population. The global health/QoL was reported to be slightly impaired in the MPN participants compared to the general population, whereas the difference between the MPN subgroups was trivial. MPN participants reported a slightly healthier lifestyle than the general population. Although there was a statistically significant difference in nearly every HRQoL aspect when the MPN subgroups were compared and when the MPN subgroups were compared to the general population, only a few are considered clinically significant [38,39,40]. 

In an international study by Scherber et al., the difference in HRQoL between the ET and the PV participants, respectively, and the general population was trivial, whereas the difference between the MF participants and the general population was small to moderate [26]. In an English study by Andersson et al., the differences in symptom burden and QoL between a MPN population and general population were more significant [14]. However, in the MPNhealthSurvey and in the study by Scherber et al. the EORTC QLQ C-30 was used to compare HRQoL in the MPN population with the general population, and in the study by Anderson et al. the MPN-SAF was used for this purpose, and the comparison of results is challenged by these different instruments used for measuring HRQoL. An explanation for the small difference in HRQoL between the MPN population in the MPNhealthSurvey and the general population might be that the patients are successfully treated. However, in a Swedish study by Abelsson et al., the symptom burden in newly diagnosed MPN patients measured by MPN-SAF in general were at equal levels as those in the MPNhealthSurvey [19]. This may indicate that it is actually questionable if current available medical treatments reduce symptom burden to a significant degree in the majority of MPN patients. However, we have to take into account the differences in populations in the study by Abelsson et al. and the MPNhealthSurvey. Furthermore, despite the recent diagnosis, MPN patients in the Swedish study might receive medical treatment at the time for the questionnaire survey, which could reduce the symptom burden compared to newly diagnosed MPN patient for whom medical treatment has not been initiated. 

In the MPNhealthSurvey the differences in symptom burden across the MPN subgroups were trivial. In an American study by Mesa et al. the MF patients had the highest and the ET patients had the lowest symptom burden and the differences between the subgroups were small [8]. In the Swedish study by Abelsson et al., the PV patients had the highest symptom burden and the differences between the subgroups were small [19]. 

The MPNhealthSurvey revealed that fatigue was highly prevalent in each of the MPN subgroups as measured by MPN-SAF. This concurs with results from an American study by Scherber et al. [13], and the English study by Anderson et al. [14]. 

In the MPNhealthSurvey, sexual problems were reported to be prevalent and burdensome. The prevalence and burden of sexual problems were in line with those presented from an international study by Geyer et al. [9]. 

BMI was significantly lower in the MPN population compared to the general population with the exception of MF participants. The small number of MF participants in the MPNhealthSurvey is a limiting factor for reliable interpretation. The MPN population did not have significantly more smokers than the general population. Previous studies, however, have found significantly more smokers among MPN patients [41,42,43]. Smoking has been proposed as a contributing factor for the development of MPNs, substantiated by a Danish case–control study [43]. Interestingly, there were more ex-smokers in the MPNhealthSurvey population compared to the general population. Knowledge of the increased risk of thrombosis in MPN patients might have been a motivating factor to quit smoking. 

Adaptation to changes in HRQoL and psychological reframing of expectations to HRQoL because of MPN as well as temporal distance to the life without MPN might influence the perceived HRQoL [38,39,40,44]. Comparison of HRQoL in the MPN population in this survey and HRQoL in the general population might be challenged by shift in perceived HRQoL among these chronic MPN patients to some extent.

Differences in HRQoL between the MPNhealthSurvey population and MPN populations in other surveys e.g., those mentioned previously might at least partly be explained by strengths and limitations in the MPNhealthSurvey. Strengths and limitations of the MPNhealthSurvey that have been described in detail in the previously published article with regard to survey design and the characteristics of respondents and nonrespondents are briefly outlined here [30]. This is the first nationwide and population-based HRQOL survey of MPN patients, which was made possible owing to the NPR and CPR [5,6,7,8,9,10,11,12,13,14,15,16,17,18,19,20,21,22,23,24,25,26,27,28,29]. The mixed-mode approach to data collection ensured that skilled use of the Internet was not a necessity for participation. To the extent of our knowledge, the Philadelphia-negative MPN population is the largest ever that has been investigated for HRQoL in one survey [5,6,7,8,9,10,11,12,13,14,15,16,17,18,19,20,21,22,23,24,25,26,27]. To the extent of our knowledge, this is also the first survey of HRQoL in MPN-U patients. The fact that the response rate was 61% and that patients of younger age, living alone, and with a lower educational level were underrepresented challenges the generalizability of the findings in this survey. The number of MF participants was only 83, and thus the representativeness of this group is not convincing. The investigation of HRQoL was comprehensive, and to the best of our knowledge, no previous HRQoL study was as comprehensive as the MPNhealthSurvey [5,6,7,8,9,10,11,12,13,14,15,16,17,18,19,20,21,22,23,24,25,26,27]. Another strength is the adjustment for the possible confounders age, sex, comorbidity, and lifestyle when comparing the HRQoL across the MPN subgroups, the adjustment for the possible confounders age and sex when comparing CCI and lifestyle across the MPN subgroups as well as comparing the MPN subgroups with the age- and sex matched general population with regard to lifestyle. Duration of disease was not taken into account in the HRQoL analyses. Duration of disease might have a significant impact on HRQoL. An unknown amount of MPN patients seem to be diagnosed with MPN years after the actual disease appearance and this would complicate both investigation of HRQoL related to disease duration and adjustment of HRQoL for duration of disease as a confounder. The NPR is generally found to be valid [28]; however, because it is based on reports from hospitals, some misclassified diagnoses are supposed to be included. Surprisingly many patients had a MPN-U diagnosis according to the NPR. Some patients registered in the NPR with an MPN-U diagnosis may be wrongly registered and actually have an ET, PV, or MF diagnosis. Surprisingly few patients were registered with an MF diagnosis in the NPR. Until 2012, MF was coded as MPN-U according to ICD-10. From 2012, MF was coded as MF. It may be that some MF patients’ diagnosis codes were not recoded in 2012. This may, to some extent, explain the remarkably impaired HRQoL in MPN-U participants. In addition, ET may develop into PV and MF, and PV into MF because of disease progression [2,3,4,45,46,47]. It is possible that some of the MPN patients who were registered in the NPR with a diagnosis of ET or PV later developed MF, but this change was not registered. If so, the symptom burden may be falsely high for those registered as ET and PV participants compared to MF participants. The MPN-SAF questionnaire has been translated into Danish according to international guidelines for translation of PRO questionnaires, but the questionnaire has not been validated in Danish [48]. A validation could potentially have led to linguistic optimizations. The survey was extensive, and the large number of questions might have discouraged some patients from participating. In addition, patients with symptoms might have been more interested in participating than patients without symptoms, and some patients with a very severe symptom burden might not have had sufficient physical and mental energy to complete the survey. The comparison of the results from the MPNhealthSurvey with those of other studies is not straightforward. This survey was a nationwide, population-based survey carried out in Denmark, whereas the participants in some studies used for comparison were from another country and from different countries based on an international collaboration in others. The differences in study populations may be reflected in the HRQoL. Finally, WHO criteria for the MPN diagnosis and treatment recommendations have changed after the MPNhealthSurvey was performed in 2013 [49]. Ruxulitinib has been introduced as treatment for myelofibrosis based on results from the COMFORT studies, and Ruxulitinib has been shown to improve HRQoL for MF patients [11]. Changes in WHO criteria for the MPN diagnosis and treatment recommendations might to some extent give a different outcome of a HRQoL survey similar to the MPNhealthSurvey performed today compared to the survey performed in 2013.

We believe that insight into Philadelphia-negative MPN patients HRQoL help understanding these chronic cancer patients’ health conditions. Insight into MPN patients’ functioning and fatigue burden compared to the general population is, for example, of importance understanding to which extent they are capable of performing in the labor market. The minor differences in HRQoL between the MPN subgroups also are important in this respect. We suggest attention is given to ET patient symptom burden [50]. Relatively many ET patients are of younger age and they are, therefore, expected to work on a daily basis in the labor market, as well as to take care of a family, and this may be difficult for them because of health issues. In an American study by Mesa et al., many ET patients reported that their disease caused reduced productivity at work, and they often had to call in sick [8]. A future study should investigate the ability of MPN patients to stay employed. MPN-U participants reported a higher symptom burden and a higher burden of mental fatigue than the other MPN subgroups. Their level of functioning was the same as that of MF participants. We assume that some of the participants in the MPNhealthSurvey registered with an MPN-U diagnosis in the NPR are actually MF patients and some are patients with prefibrotic primary myelofibrosis who were registered in the NPR or diagnosed with an MPN-U diagnosis by mistake. However, it might also be, that MPN-U patients actually have symptom burden as high as, or even higher than, that of MF patients. Previous research on sexuality in MPN patients used the MPN-SAF questionnaire that has one question on presence and severity of sexual problems [7,9,14,17,19,26]. Further investigation of the nature of the sexual problems MPN patients struggle with may highlight potential areas for medical and psychological intervention [9]. This survey found relatively many participants to be physically active. In the study by Scherber et al., physical activity was found to reduce fatigue [13]. In the study by Tolstrup et al. based on the data from the MPNhealthSurvey a positive association between physical activity and QoL was found [31]. Considering these findings, we suggest physical activity to be studied as a rehabilitation initiative for MPN patients to hopefully improve physical functioning, reduce fatigue, and improve QoL. Lifestyle changes in general seem appealing to these chronic cancer patients and it may be considered relevant to support these in the hematological outpatient clinics in order to reduce the risk of cardiovascular complications and comorbidities, and to improve HRQoL [13]. 

## 4. Materials and Methods

The survey design of the MPNhealthSurvey has been described in a previously published article [30]. 

### 4.1. Participants

Individuals with a diagnosis of ET, PV, MF, or MPN-U recorded in the NPR in Denmark, ≥16 years of age, and alive on September 4, 2013, were invited to participate in the survey. 

### 4.2. Data Collection

The MPN patients’ postal addresses were retrieved from the CPR. A postal letter was sent to everyone who met the inclusion criteria. The participants could either complete and return the enclosed survey booklet or submit their answers to the survey questions online. 

### 4.3. Questionnaires and Additional Questions

HRQoL investigation using the following validated questionnaires is presented here: (1) SF-36 [34]; (2) EORTC QLQ-C30 [35]; (3) MPN-SAF which is used together with BFI [26,36]; (4) the MPN-SAF Total Symptom Score (MPN-SAF TSS) which is an abbreviated symptom score developed from the MPN-SAF and BFI [23,36]; (5) MFI [37]. Additional questions covered lifestyle. 

### 4.4. Baseline Demographics, Confounders, and General Population Data

We obtained information on age, sex, and ethnicity from the CPR and on living arrangement, education, and employment from Statistics Denmark’s register [51]. Information on comorbid diagnoses was obtained from the NPR, and the Charlson Comorbidity Index (CCI) was used to assess the participants’ comorbidity burden [28,52,53]. 

While investigating differences in HRQoL among participants with different MPN subtypes, we adjusted for age, sex, CCI, and lifestyle. 

The lifestyle data from a health and morbidity survey of the general population in Denmark conducted in the spring in 2013 by The National Institute of Public Health served as reference data [54]. The general population was age- and sex-matched to the MPN population. Additionally, Danish population-based reference data for EORTC QLQ C-30 was used [55]. The EORTC QLQ C-30 survey on the general Danish population was conducted from October 2012 to March 2013.

### 4.5. Statistics

Characteristics of respondents and nonrespondents were compared using logistic regressions, except for age and CCI, for which linear regressions were used. Regression models were used to investigate differences in HRQoL between MPN subgroups and between MPN subgroups and the general population: for the SF-36, a gamma regression model was used to account for the skewed distribution of responses. For the EORTC QLQ C-30, linear regression of log-transformed scores accounted for the non-normal distribution of responses. Linear regression of log-transformed scores was also used for the BFI and the MPN-SAF to account for non-normal distribution of responses in addition to the zero-inflated Poisson-regression for individual items to account for an inflated number of ‘0’ responses. Finally, for the MFI, a Poisson regression model was applied to account for the non-normal distribution of responses. The average EORTC QLQ C-30 scores for the MPN subgroups were compared with weighted general population averages, in order to ensure that the population average reflected the age and sex distribution in the subgroups with different MPN subtypes. Comparison analyses were conducted using two-sided t-tests. A significance level of 0.05 was used. 

## 5. Conclusions

A total of 2228 Philadelphia-negative MPN patients participated in the MPNhealthSurvey in Denmark. Overall, the MPN participants reported their HRQoL to be inferior to the general population but the difference was minor, and the differences in HRQoL across groups of participants with different MPN subtypes were subtle. Fatigue and sexual problems were prevalent and burdensome. MPN participants reported a slightly healthier lifestyle than the general population.

## Figures and Tables

**Table 1:** Characteristics of respondents and nonrepondents.

	Respondents (Participants)	Nonrespondents	*p*-Value
	ET+PV+MF+MPN-U	ET+PV-MF+MPN-U	
Total (n,%)	2228 (61%)	1428 (39%)	
Age (mean, SD)	70.7 (14.6)	69.2 (12.4)	<0.001 *
Sex (n,%)			>0.1 **
Female	1274 (57%)	819 (57%)	
Male	954 (43%)	609 (43%)	
Female (n,%)			<0.0001 ***
20–29 years old	6 (<1%)	5 (<1%)	
30–39	24 (2%)	19 (2%)	
40–49	75 (6%)	54 (7%)	
50–59	171 (13%)	87 (11%)	
60–69	324 (25%)	155 (19%)	
70–79	403 (32%)	192 (23%)	
80–89	238 (19%)	226 (28%)	
≥90	33 (3%)	81 (10%)	
Male (n,%)			0.0019 ***
20–29 years old	4 (<1%)	2 (<1%)	
30–39	16 (2%)	15 (3%)	
40–49	46 (5%)	44 (7%)	
50–59	133 (14%)	100 (16%)	
60–69	267 (28%)	158 (26%)	
70–79	323 (34%)	159 (26%)	
80–89	150 (16%)	107 (18%)	
≥90	15 (1%)	24 (4%)	
Living arrangement (n,%)			<0.0001 ***
Living alone	737 (33%)	723 (51%)	
Living with someone	1475 (66%)	659 (46%)	
Missing	16 (1%)	46 (3%)	
Education (n,%)			<0.0001 ***
Basic school	677 (30%)	610 (43%)	
Upper secondary/	951 (43%)	478 (33%)	
vocational school			
Higher education	521 (23%)	215 (15%)	
Missing	79 (4%)	125 (9%)	
Employment (n,%)			0.0076 ***
Employed	601 (27%)	319 (22%)	
Unemployed	51 (2%)	42 (3%)	
Retired	1575 (71%)	1067 (75%)	
Missing	1	0	
Ethnicity (n,%)			<0.0001 ***
Danish/Western	2125 (95%)	1264 (89%)	
Non-Western	87 (4%)	118 (8%)	
Missing	16 (1%)	46 (3%)	
Duration of disease (n,%)			0.0259 ***
<1 year	157 (7%)	78 (6%)	
1–4 years	792 (36%)	476 (33%)	
≥5 years	1279 (57%)	874 (61%)	
Comorbidity			
CCI (mean, SD)	1.5 (2.0)	1.1 (1.7)	<0.0001 ***
CCI score (n,%)			
ET			
0	436 (54%)	250 (46%)	<0.0001 ***
1–2	286 (35%)	189 (34%)	
≥3	92 (11%)	109 (20%)	
PV			
0	435 (48%)	245 (41%)	0.0014 ***
1–2	336 (37%)	233 (38%)	
≥3	139 (15%)	125 (21%)	
MF			
0	42 (51%)	9 (23%)	0.0024 ***
1–2	21 (25%)	12 (31%)	
≥3	20 (24%)	18 (46%)	
MPN–U			
0	193 (46%)	89 (37%)	0.0009 ***
1–2	171 (41%)	86 (36%)	
≥3	57 (13%)	63 (27%)	
MPN subtype (n,%)			0.06 ***
ET	814 (36%)	548 (38%)	
PV	910 (41%)	603 (42%)	
MF	83 (4%)	39 (3%)	
MPN-U	421 (19%)	238 (17%)	

CCI: Charlson Comorbidity Index. * Adjusted for sex; ** Adjusted for age; *** Adjusted for sex and age.

**Table 2:** Differences in health-related quality of life (HRQoL) between participants with different MPN subtypes and differences in HRQoL between participants and the general population.

Questionnaire Scales	Questionnaire Scores Mean, SD									
	General Population	ET	*p*-Value *	PV	*p*-Value *	MF	*p*-Value *	MPN-U	*p*-Value *	*p*-Value **
**SF-36**										
Physical component summary measure		47.7 (9.9)		46.5 (9.7)		45.0 (10.5)		45.6 (10.0)		<0.001
Mental component summary measure		51.5 (9.9)		51.3 (10.0)		51.9 (9.3)		50.7 (9.9)		0.002
**EORTC QLQ C-30**										
Global health status/QoL	73 (23)	68.5 (23.9)	<0.001	67.1 (24.5)	<0.001	67.6 (26.5)	0.036	64.9 (24.6)	<0.001	0.002
Functional scales										
physical functioning	86 (20)	77.2 (22.3)	<0.001	73.7 (22.6)	<0.001	70.0 (24.5)	<0.001	71.8 (23.3)	<0.001	<0.001
role functioning	84 (27)	76.5 (28.3)	<0.001	75.1 (28.7)	<0.001	71.1 (32.7)	<0.001	72.4 (29.8)	<0.001	0.026
emotional functioning	84 (19)	79.8 (21.3)	<0.001	79.6 (21.1)	<0.001	81.5 (19.5)	0.091	78.4 (21.9)	<0.001	<0.001
cognitive functioning	87 (20)	79.9 (22.8)	<0.001	79.4 (22.5)	<0.001	77.4 (22.7)	<0.001	78.9 (21.8)	<0.001	0.067
social functioning	90 (21)	83.9 (24.7)	0.010	83.1 (24.1)	<0.001	81.9 (24.3)	0.005	82.7 (25.2)	<0.001	<0.001
Symptoms scales										
fatigue	24 (23)	34.0 (25.8)	<0.001	35.9 (25.5)	<0.001	38.5 (26.1)	<0.001	37.4 (25.0)	<0.001	0.009
nausea/vomiting	3 (10)	6.2 (13.7)	<0.001	6.2 (14.0)	<0.001	4.7 (10.9)	0.106	6.0 (13.7)	<0.001	0.045
pain	20 (26)	22.9 (27.1)	0.017	23.3 (26.5)	0.005	23.0 (25.7)	>0.1	24.8 (28.2)	0.006	<0.001
dyspnea	11 (21)	20.3 (26.8)	<0.001	21.1 (27.0)	<0.001	25.1 (29.2)	<0.001	21.5 (27.8)	<0.001	<0.001
insomnia	21 (27)	29.4 (31.7)	<0.001	30.4 (32.4)	<0.001	22.5 (26.7)	>0.1	31.1 (31.9)	<0.001	0.009
appetite loss	7 (18)	11.9 (23.5)	<0.001	13.7 (25.5)	<0.001	21.5 (32.5)	<0.001	16.6 (26.5)	<0.001	0.004
constipation	6 (17)	13.7 (24.6)	<0.001	12.3 (23.3)	<0.001	14.4 (23.5)	0.001	12.5 (22.7)	<0.001	>0.1
diarrhea	7 (17)	14.4 (24.4)	<0.001	14.4 (24.5)	<0.001	16.9 (25.3)	<0.001	14.4 (25.6)	<0.001	0.046
financial difficulties	6 (18)	8.1 (19.9)	0.005	10.0 (23.7)	<0.001	12.3 (24.4)	0.016	9.6 (22.4)	0.004	0.008

SF-36 (Short Form Health Survey 36): The scales range in a score from 0 to 100. A high component summary score measure is the most favorable. EORTC QLQ C-30 (European Organisation for Research and Treatment of Cancer Quality of Life Questionnaire Core-30): All of the scale measures range in a score from 0 to 100. A high score for a functional scale represents a high/healthy level of functioning. A high score for a symptom scale represents a high level of symptomatology. A high score for the global health/QoL represents a high HRQoL. * Difference between the MPN subgroup and the general population. ** Difference between the MPN subgroups. Adjusted for age, sex, comorbidity (CCI), and lifestyle (BMI, smoking, alcohol consumption, physical activity)

**Table 3:** Symptoms, symptom burden, and QoL. Differences between participants with different MPN subtypes.

MPN-SAF	Questionnaire Score Mean, SD, Prevalence%				
	ET	PV	MF	MPN-U	Difference in Mean Symptom Burden between MPN Subtypes *p*-Value *
Total symptom score (TSS)	17.5 (14.9)	17.8 (15.1)	16.2 (12.7)	21.1 (16.1)	<0.001
fatigue (BFI score)	2.5 (2.3) 82%	2.6 (2.4) 82%	2.8 (2.6) 74%	2.8 (2.3) 87%	<0.001
early satiety	2.0 (2.4) 56%	1.7 (2.3) 53%	2.5 (3.6) 46%	2.1 (2.5) 56%	<0.001
abdominal pain	1.2 (2.0) 36%	0.9 (1.6) 37%	0.9 (1.1) 55%	1.0 (1.7) 40%	0.021
abdominal discomfort	1.9 (2.4) 53%	1.4 (2.0) 51%	1.4 (1.3) 64%	1.5 (1.9) 54%	0.004
inactivity	1.9 (2.3) 54%	1.9 (2.2) 57%	2.1 (2.3) 73%	2.4 (2.4) 62%	<0.001
headaches	1.6 (2.3) 50%	1.7 (2.3) 50%	1.3 (1.8) 46%	1.8 (2.1) 56%	0.004
concentration problem	1.9 (2.6) 49%	2.1 (2.5) 58%	2.5 (3.0) 64%	2.4 (2.7) 58%	0.011
dizziness	2.0 (2.3) 60%	2.2 (2.5) 64%	2.0 (1.8) 73%	2.2 (2.2) 72%	0.002
numbness	2.1 (2.8) 51%	2.1 (2.7) 53%	0.7 (1.2) 46%	2.0 (2.3) 60%	0.013
insomnia	2.5 (2.8) 64%	2.5 (2.8) 62%	1.4 (1.7) 55%	2.6 (2.6) 66%	<0.001
sad mood	2.0 (2.4) 59%	2.1 (2.5) 57%	1.6 (1.7) 73%	2.4 (2.7) 66%	<0.001
sexuality problems	3.3 (3.5) 62%	3.3 (3.3) 64%	3.8 (3.3) 82%	3.8 (3.9) 62%	<0.001
cough	1.4 (2.2) 42%	1.4 (2.2) 44%	1.3 (1.8) 46%	1.9 (2.4) 52%	<0.001
night sweats	2.4 (2.7) 63%	2.4 (2.6) 67%	2.5 (2.5) 64%	3.6 (3.1) 70%	0.046
itching	1.7 (2.4) 50%	2.4 (2.9) 59%	0.5 (1.0) 18%	2.2 (2.7) 52%	<0.001
bone pain	1.5 (2.6) 39%	1.8 (2.6) 48%	0.9 (1.4) 46%	1.9 (2.5) 54%	<0.001
fever (>100F)	0.4 (1.4) 17%	0.3 (0.8) 17%	0.2 (0.6) 9%	0.4 (1.0) 18%	<0.001
unintentional weight loss last 6 months	0.6 (1.8) 17%	0.7 (1.7) 22%	1.4 (2.9) 27%	1.2 (2.4) 28%	<0.001
quality of life	2.5 (2.3) 74%	2.4 (2.2) 76%	2.1 (1.8) 73%	2.7 (2.2) 78%	<0.001

MPN-SAF: Myeloproliferative Neoplasm Symptom Assessment Form. The symptoms range in a number from 0 to 10. A higher number indicates high symptomatology or impaired QoL. The total symptom score ranges from 0 to 100. A high number indicates high symptomatology and impaired QoL. * Adjusted for age, sex, comorbidity (CCI), and lifestyle (BMI, smoking, alcohol consumption, physical activity).

**Table 4:** Characterization of fatigue. Differences between participants with different MPN subtypes.

Questionnaire Scales	Questionnaire Scores Mean, SD				
	ET	PV	MF	MPN_U	*p*-Value *
MFI					
general fatigue	11.5 (4.9)	11.6 (4.9)	12.4 (5.1)	12.0 (4.5)	<0.001
physical fatigue	11.3 (4.9)	12.0 (4.9)	12.2 (5.6)	12.1 (4.6)	<0.001
reduced activity	10.1 (4.7)	10.9 (4.9)	11.1 (5.6)	11.2 (4.7)	<0.001
reduced motivation	8.3 (3.6)	8.8 (3.7)	8.6 (3.9)	8.8 (3.6)	<0.001
mental fatigue	8.6 (4.2)	8.7 (4.3)	8.2 (4.5)	8.9 (4.2)	<0.001

MFI: Multidimensional Fatigue Inventory. All of the scales measure range in a score from 4 to 20. A high score indicates a high level of fatigue. * Adjusted for age, sex, comorbidity (CCI), lifestyle (BMI, smoking, alcohol consumption, physical activity).

**Table 5:** Comorbidity and lifestyle. Differences in lifestyle between the MPN subgroups and the general population. Differences in comorbidity and lifestyle between participants with different MPN subtypes.

	ET	GP	*p*-Value *	PV	GP	*p*-Value *	MF	GP	*p*-Value *	MPN-U	GP	*p*-Value *	*p*-Value **
Comorbidity (n,%)													<0.0001
CCI = 0	436 (54%)			435 (48%)			42 (51%)			193 (46%)			
CCI = 1 or 2	286 (35%)			336 (37%)			21 (25%)			171 (41%)			
CCI ≥ 3	92 (11%)			139 (15%)			20 (24%)			57 (13%)			
BMI (n,%)			0.0011			0.0004			>0.1			0.029	>0.1
BMI < 18.5	19 (2%)	400 (3%)		24 (3%)	383 (2%)		2 (2%)	27 (2%)		14 (3%)	234 (3%)		
18.5 ≤ BMI < 25.0	425 (55%)	7166 (47%)		446 (51%)	7580 (45%)		39 (51%)	702 (46%)		208 (52%)	3545 (45%)		
25.0 ≤ BMI < 30.0	242 (31%)	5460 (36%)		285 (32%)	6639 (39%)		27 (35%)	581 (38%)		123 (31%)	2953 (38%)		
BMI ≥ 30	95 (12%)	2194 (14%)		124 (14%)	2421 (14%)		11 (14%)	212 (14%)		57 (14%)	1127 (14%)		
Missing	33 (4%)			31 (3%)			4 (5%)			19 (5%)			
Smoking (n,%)			>0.1			0.05			>0.1			>0.1	>0.1
Yes	152 (19%)	2880 (19%)		152 (17%)	3380 (20%)		12 (15%)	280 (18%)		82 (20%)	1494 (19%)		
Ex-smoker	340 (43%)	6203 (40%)		409 (47%)	7378 (42%)		39 (49%)	653 (42%)		179 (44%)	3340 (42%)		
No	301 (38%)	6394 (41%)		317 (36%)	6546 (38%)		29 (36%)	612 (40%)		144 (36%)	3089 (39%)		
Missing	21 (3%)			32 (4%)			3 (4%)			16 (4%)			
Alcohol (n,%)			>0.1			0.01			>0.1			0.0017	0.0314
units ≤ 7	461 (64%)	7328 (61%)		518 (63%)	7811 (57%)		41 (58%)	691 (58%)		263 (71%)	3710 (62%)		
8 ≤ units ≤ 14	154 (21%)	2585 (22%)		161 (20%)	3130 (23%)		16 (23%)	273 (23%)		53 (15%)	1292 (21%)		
15 ≤ units ≤ 21	62 (9%)	1036 (9%)		66 (8%)	1307 (10%)		4 (5%)	110 (9%)		27 (7%)	527 (9%)		
22 ≤ units	47 (6%)	983 (8%)		76 (9%)	1340 (10%)		10 (14%)	118 (10%)		26 (7%)	507 (8%)		
Missing	90 (11%)			89 (10%)			12 (14%)			52 (12%)			
Physical activity (n,%)			<0.0001			<0.0001			0.004			<0.0001	0.0032
Hard training and	53 (7%)	187 (1%)		37 (4%)	177 (1%)		0	27 (2%)		16 (4%)	84 (1%)		
competitive sports													
several times a week													
Training, heavy garden work	168 (21%)	2230 (15%)		181 (21%)	2500 (15%)		16 (23%)	219 (14%)		67 (17%)	1012 (13%)		
or similar ≥ 4 times a week													
Walk, bicycle, light garden	377 (49%)	9251 (61%)		423 (50%)	10368 (61%)		27 (38%)	882 (58%)		195 (50%)	4728 (60%)		
work or similar≥ 4 times													
a week													
Read, watch TV, or	177 (23%)	3559 (23%)		210 (25%)	4031 (23%)		28 (39%)	403 (26%)		114 (29%)	2006 (26%)		
other sedentary work													
Missing	39 (5%)			59 (6%)			12 (14%)			29 (7%)			

CCI: Charlson Comorbidity Index. BMI: Body Mass Index. GP: General population. * Difference between the MPN subgroup and age- and sex-matched general population. ** Difference between the MPN subgroups. Adjusted for age and sex.
